# Unraveling the Formation of Ternary AgCuSe Crystalline Nanophases and Their Potential as Antibacterial Agents

**DOI:** 10.1021/acs.chemmater.4c01604

**Published:** 2024-10-09

**Authors:** Mengxi Lin, Beatriz Vargas, Lluís Yedra, Heleen van Gog, Marijn A. van Huis, Rafael G. Mendes, Jordi Llorca, Manel Estruch-Blasco, Manuel Pernia Leal, Eloísa Pajuelo, Sònia Estradé, Francesca Peiró, Laura Rodríguez, Albert Figuerola

**Affiliations:** † Department of Inorganic and Organic Chemistry, Inorganic Chemistry Section, Universitat de Barcelona, Carrer de Martí i Franquès, 1-11, 08028 Barcelona, Spain; ‡ Institute of Nanoscience and Nanotechnology (IN2UB), Universitat de Barcelona, Carrer de Martí i Franquès, 1-11, 08028 Barcelona, Spain; § Laboratory of Electron Nanoscopies (LENS-MIND), Department of Electronics and Biomedical Engineering, Universitat de Barcelona, C/Martí i Franquès 1, 08028 Barcelona, Spain; ∥ Nanostructured Materials and Interfaces, Zernike Institute for Advanced Materials, University of Groningen, Nijenborgh 4, 9747 AG Groningen, The Netherlands; ⊥ Soft Condensed Matter, Debye Institute for Nanomaterials Science, Utrecht University, Princetonplein 5, 3584 CC Utrecht, The Netherlands; # Institute of Energy Technologies, Department of Chemical Engineering and Center for Research in Multiscale Science and Engineering, Universitat Politècnica de Catalunya, EEBE, Eduard Maristany 10-14, 08019 Barcelona, Spain; ∇ Departamento de Química Orgánica y Farmacéutica, Facultad de Farmacia, Universidad de Sevilla, c/Profesor García González, 2, 41012 Sevilla, Spain; ○ Departamento de Microbiología y Parasitología, Facultad de Farmacia, Universidad de Sevilla, c/Profesor García González, 2, 41012 Sevilla, Spain

## Abstract

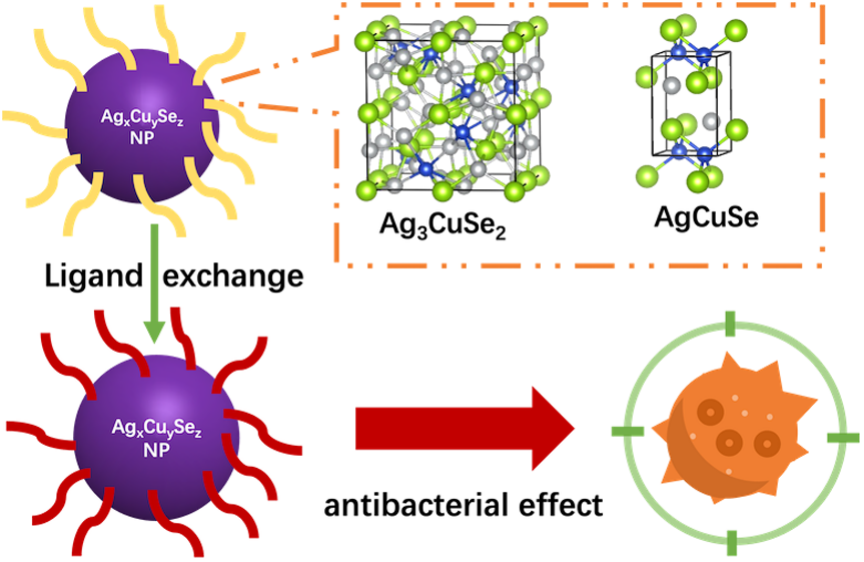

AgCuSe nanoparticles could contribute to the growth of strongly light-absorbing thin films and solids with fast ion mobility, among other potential properties. Nevertheless, few methods have been developed so far for the synthesis of AgCuSe nanoparticles, and those reported deliver nanostructures with relatively large sizes and broad size and shape distributions. In this work, a colloidal cation exchange method is established for the easy synthesis of AgCuSe NPs with ca. 8 nm diameters and narrow size dispersion. Notably, in this lower size range the conucleation and growth of two stoichiometric ternary compounds are generally observed, namely the well-known *eucairite* AgCuSe compound and the novel *fischesserite*-like Ag_3_CuSe_2_ phase, the latter being less thermodynamically stable as predicted computationally and assessed experimentally. An optimal range of Cu/Ag precursor molar ratio has been identified to ensure the growth of ternary nanoparticles and, more specifically, that of the metastable Ag_3_CuSe_2_ nanophase isolated for the first occasion. The attained size range for the material paves the way for utilizing AgCuSe nanoparticles in new ways within the field of biomedicine: the results obtained here confirm the antibacterial activity of the new Ag_*x*_Cu_*y*_Se_*z*_ nanoparticles against Gram-positive bacteria, with significantly low values of the minimal inhibitory concentration.

## Introduction

The fabrication and development of diverse metal chalcogenide nanomaterials (NMs) represent an ongoing pursuit driven by their advantageous properties, making them well suited for a broad spectrum of applications, particularly in the field of energy conversion, including thermoelectric,^1−4^ solar cell light harvesting,^5,6^ catalysis,^7−9^ and display technologies.^10−12^ The widespread utility of metal chalcogenide NMs can be attributed to their highly tunable optoelectronic properties, which can be accessed through synthetic control over the composition and morphology (size and shape) of a single nanoparticle (NP).^13,14^ In the case of obtaining ternary/multinary NPs in colloidal chemistry, where the desired property can be further enhanced or even diversified, a third element (mostly a second metal cation) is required, which can be added either directly into reaction mixtures along with other element precursors (direct synthesis)^15^ or into presynthesized homogeneously distributed binary NPs (postsynthesis).^16^ Here, the cation exchange (CE) technique is an indispensable postsynthetic method for delivering the final NPs with remarkable control over morphology, stoichiometry, and arrangement of atomic positions, compared to those NPs obtained through direct synthesis.^17,18^ Furthermore, this method could also play a role in stabilizing metastable crystallographic phases in some cases.^19,20^

Coinage metal chalcogenide nanomaterials are fast emerging as materials of choice for a wide range of applications (thermo-electrics, photovoltaic solar cells, memory devices, nonlinear optics, energy storage, etc.) due to their various physical properties such as low band gaps of 0.15–2.0 eV, low toxicity as compared to that of Pb- and Cd-containing chalcogenides, high absorption coefficients, NIR emission, etc.^21−24^ In particular, ternary AgCu-based chalcogenides have drawn researchers’ attention due to their high electron mobility, low thermal conductivity, cationic order–disorder transitions during phase change, and fast ionic conductivity, which make them potentially interesting as thermoelectric and electrochemically active materials.^23−28^ Currently, only one AgCuSe stoichiometric material is known, which adopts two different polymorphs: a low-temperature β-phase, described either as a *tetragonal* or *orthorhombic* structure, and a high-temperature α-phase with a *cubic* structure, both of them favoring high mobility of the ions, and therefore, offering great promise in thermoelectric and electrochemical devices (batteries, fuel cells, gas sensors, etc.).^23,24^

Concerning the synthesis of nanometric forms of AgCuSe, three synthetic strategies have been reported thus far. The first documented method for producing AgCuSe NPs involves a direct synthesis strategy. In this approach, precursor salts for both copper and silver are simultaneously introduced into a solution containing elemental selenium, in the presence of NaBH_4._^24^ The second reported method employs a thermolysis process, wherein metallomesogen compounds serve as templates.^29^ The resulting AgCuSe NPs produced by both of these methods consistently display a broad distribution in both size and shape. This variability poses a challenge to establishing a meaningful correlation between their internal morphologies and their exhibited properties, which is of critical importance for their applications. The only reported well-controlled e.g., monodispersed AgCuSe NPs were obtained by Eikey et al. in 2020.^30^ They achieved this by employing a CE reaction between Cu_2–*x*_Se NPs and AgNO_3_ solution, resulting in the formation of single-phase and homogeneous 53 nm AgCuSe NPs, which constituted over 77% of the sample population. Prior to this, Poudeu et al. were the first ones who successfully produced AgCuSe nanoplatelets by the CE reaction by using a similar approach in 2015.^31^ The AgCuSe nanoplatelets obtained in this instance ranged in size from 0.5 to 3.0 μm. A CE approach performed on smaller and homogeneous precursor NPs could help to fully transform initial binary crystals into ternary NPs of interest, avoiding the presence of nonternary impurities in the final samples, while preserving the suitable size and shape distribution of the precursor nanostructures.

Here, we report a modified CE reaction for the synthesis of ternary AgCuSe NPs. The CE reaction starts from Ag_2_Se NPs as precursors instead of Cu_2-x_Se NPs, and leads to homogeneously distributed 8 nm ternary NPs. Additionally, the Cu(I) phosphane complex is used as a guest cation source due to its higher solubility in apolar solvents compared to conventionally used metal salts. The results obtained show the formation of two different stoichiometric ternary phases, namely, the well-known *orthorhombic* AgCuSe and the new *cubic* Ag_3_CuSe_2_. Their relative thermodynamic stability was studied by density functional theory (DFT) calculations and also assessed experimentally. These ternary materials show a promising antibacterial activity, higher than that recently observed for pure Ag NPs, as concluded from our experiments with Gram-positive bacteria.

## Experimental Section

### Chemicals

Silver chloride (AgCl, 99.9%), selenium powder (Se, 99.9%), and tri-*n*-octylphosphine (TOP, 97%) were obtained from Strem Chemicals. Oleylamine (OLAm, 70%), tri-*n*-octylphosphine oxide (TOPO, 99), triphenylphosphine (PPh_3_, 99%), copper(I) iodide (CuI, ≥99.5%), and toluene (99.9%) were purchased from Sigma-Aldrich. Ethanol (EtOH, 96%) and acetone (99.5%) were obtained from Panreac. Acetonitrile (MeCN, ≥99%) was purchased from VWR. AuClPPh_3_ was synthesized by our collaboration group. The CuIPPh_3_ compounds were obtained using a previously reported method by Espinet and co-workers.^32^

#### Synthesis of Ag_2_Se NPs

The synthesis of Ag_2_Se NPs is based on the procedure published by Sahu and co-workers^33^ with some modifications. First, two precursor solutions were prepared in the glovebox: 474 mg (6 mmol) of Se was dissolved in 6 mL of TOP, and 572 mg (4 mmol) of AgCl was dissolved in 4 mL of TOP. Next, a reaction mixture composed of 7.8 g of TOPO and 6.6 mL of OLAm was degassed under vacuum at 120 °C for 30 min. Afterward, the temperature was raised to 180 °C under a N_2_ atmosphere, followed by an injection of Se-TOP. As soon as the temperature was back to 180 °C, a second injection of AgCl-TOP was performed. After 20 min of reaction, the solution was cooled down naturally. During the cooling-down process, 5 mL of toluene was added to the reaction mixture at around 50 °C to avoid the solidification of the solvent. Finally, the solution was washed twice with EtOH, centrifuged for 4 min at 4500 rpm, and redispersed in 4 mL of toluene.

#### Synthesis of Ag_*x*_Cu_*y*_Se_*z*_ NPs

The synthesis of Ag_*x*_Cu_*y*_Se_*z*_ NPs begins with the preparation of CuIPPh_3_-stock solution: 20 μmol (9.1 mg) of presynthesized CuIPPh_3_ was added into 20 mL of toluene, and the whole mixture was sonicated for 30 min to form the suspension. Afterward, 0.5, 2, 4, and 8 μL of CuIPPh_3_-stock solution was added quickly into each 200 μL of Ag_2_Se NP colloidal dispersion in toluene, respectively. The reaction mixture was stirred at room temperature for 2 h and washed once with EtOH. After centrifuging at 4500 rpm for 3 min, the final product was redispersed in 1 mL of toluene.

#### Synthesis of Ag_*x*_Au_w_Cu_*y*_Se_*z*_ NPs

The synthesis of Ag_*x*_Au_w_Cu_*y*_Se_*z*_ NPs is a postsynthesis based on the previously synthesized Ag_*x*_Cu_*y*_Se_*z*_ NPs. 2 μmol (0.8 mg) of presynthesized AuClPPh_3_ was added directly into 1 mL of Ag_*x*_Cu_*y*_Se_*z*_ NP dispersion, followed by 2 h stirring at room temperature. The final NPs were redispersed in 1 mL of toluene and washed with EtOH, followed by centrifugation for 3 min at 4500 rpm.

#### Functionalization of Ag_*x*_Cu_*y*_Se_*z*_ NPs for Antibacterial Tests

The ligand synthesis and intermediate compounds are described in detail in the Supporting Information.

The functionalization of Ag_*x*_Cu_*y*_Se_*z*_ NPs was performed by a ligand exchange protocol described by Pernia Leal and co-workers.^34^ In brief, a solution of compound 4 (28.7 mg, 0.017 mmol) in 2 mL of CHCl_3_ was added to a solution of Ag_*x*_Cu_*y*_Se_*z*_ NPs in toluene (2 mL, 0.180 mg of Ag) in a 12 mL glass vial. The NP suspension was placed in an ultrasound bath for 1 h. After that, it was incubated at 50 °C for 4 h. Next, the mixture was transferred into a separation funnel, and 5 mL of H_2_O, 5 mL of toluene, and 10 mL of acetone were added. The biphasic mixture was shaken, and the brown aqueous phase was collected. The organic phase was extracted again with 5 mL of H_2_O. The combined aqueous phases were placed in a rot evaporator to remove the residual organic solvents. The ligand exchanged NPs were purified by using 10 kDa cutoff centrifugal filters at 4500 rpm for 15 min until the filtrate was completely clear. The functionalized NPs were resuspended in phosphate-buffered saline (PBS) for further analysis.

#### Determination of Minimal Inhibitory Concentration (MIC) and Minimal Bactericidal Concentration (MBC)

MIC was determined by the microdilution method in 96-well microtiter plates, according to CLSI.^35^ Wells were filled with 200 μL of Müeller–Hinton (MH) broth containing decreasing concentrations of Ag_*x*_Cu_*y*_Se_*z*_ NPs (a serial dilution from 80 to 0.1 mg of Ag L^–1^), together with a control row with MH broth without NPs. Wells were inoculated with 5 μL of overnight cultures of the type strains *Escherichia coli* ATCC 25922, *Pseudomonas aeruginosa* ATCC 27853, or *Staphylococcus aureus* ATCC 25923 (in triplicate). Plates were sealed and incubated at 37 °C for 24 h. After incubation, the plates were visually inspected for turbidity. The minimal concentration of NPs in the wells where no turbidity was observed was considered as the MIC.

For the determination of MBC, 100 μL of the content of transparent wells (without turbidity) were spread on plates containing TSA medium without NPs. Plates were additionally incubated for 24 h at 37 °C, and after incubation, they were inspected for the formation of colonies. The minimal concentration of NPs in the well corresponding to a plate without colonies was considered as the MBC.

#### Observation of Biofilm Formation by *S. aureus* onto Glass Slides in the Absence and the Presence of Ag_*x*_Cu_*y*_Se_z_ NPs

Since Ag_*x*_Cu_*y*_Se_*z*_ NPs were effective mainly against *S. aureus*, the observation of cell morphology was only done with this strain. Glass surfaces of 1 cm diameter were deposited on the bottom of 24-well polystyrene plates and covered with 1 mL of TSB (tryptone soy broth) containing Ag_*x*_Cu_*y*_Se_*z*_ NPs (5 or 10 mg of Ag L^–1^), together with controls without NPs. All of the wells were inoculated with 100 μL of an overnight culture of *S. aureus* ATCC 25923 (in triplicate) and incubated for 24 h at 37 °C without shaking. After incubation, bacterial cultures were removed, and the glass disks were washed thrice with 1 mL of sterile distilled water. Bacteria attached to the glass surface were fixed for 2 h at room temperature in a solution of 2.5% glutaraldehyde dissolved in 0.2 M cacodylate buffer, pH 7.2. After fixation, glass disks were washed thrice with 0.2 M cacodylate buffer pH 7.2. Bacteria were dehydrated in acetone series (from 50% to pure acetone), frozen to a critical point at −80 °C using LEICA EM CPD 300, and sputtered with Au/Pd using equipment LEICA ACE600 for observation by SEM on a Zeiss Evo microscopy instrument (Zeiss, Germany) operated at 10 and 20 kV acceleration voltage.^36^ While the bacteria were observed, the preliminary determination of Ag accumulation was performed by scanning electron microscopy-energy-dispersive X-ray analysis (SEM-EDX).

#### Characterization Methods

X-ray diffraction (XRD) spectra were acquired with a PANalytical X′pert Pro MPD Alpha l diffractometer operating in θ/2θ geometry at 45 kV, 40 mA, and λ = 1.5406 Å (Cu Kα1). Thin layers of samples were prepared by drop casting and evaporation of the solvent in a monocrystalline Si holder of 15 mm diameter and 0.15 mm height. Scans in the range 2θ = 4–100° were run at a step size of 2θ = 0.017° and 100 s per step. The data were treated with Xpert HighScorePlus software.

The composition and concentration of the NP solutions were determined by inductively coupled plasma-atomic emission spectroscopy (ICP-AES). The measurements were carried out with an Optima 3200 RL PerkinElmer spectrometer. For those measurements, 50 μL of solutions were precipitated in MeOH and redispersed in CHCl_3_. The solution was evaporated in an oven overnight at 90 °C. Before the vial was sealed, 2.5 mL of aqua regia were added to the precipitate and then heated to 90 °C for 72 h. The resulting solution was transferred to a 25 mL volumetric flask and diluted with Milli-Q water.

Nuclear magnetic resonance (NMR) spectra were recorded on a BRUKER Avance NEO 400 and Avance III 300 MHz apparatus. Deuterated chloroform was used and is indicated in parentheses for each compound. The chemical shift values (δ) were referred to as tetramethylsilane and used as an internal reference.

Fourier transform infrared (FTIR) spectra were recorded with an Invenio-X Bruker instrument using a single reflection attenuated total reflectance (ATR) accessory (MIRacle ATR, PIKE Technologies) coupled to a liquid nitrogen-cooled mercury cadmium telluride detector. All spectra were recorded in the 4000–400 cm^–1^ range at 4 cm^–1^ resolution and accumulated 32 scans. Samples were deposited onto the diamond ATR crystal by drop casting of highly concentrated ligand or NP solutions.

The hydrodynamic (HD) size of the functionalized NPs was measured on a Zetasizer Nano ZS90 instrument from Malvern Panalytical. The measurements were performed on a cell type: ZEN0040 disposable cuvette, setting a refractive index of 0.135 and absorption of 3.990. The number of measurements was three, and the duration was set as automatic.

X-ray photoelectron spectroscopy (XPS) was carried out in a SPECS system equipped with a PHOIBOS 150 EP Hemispherical Energy Analyzer and an MCD-9 detector, using an Al Kα X-ray source of 1486.6 eV energy and 150 W power and a pass energy of 20 eV. The X-ray source is placed at 54° with respect to the analyzer axis and is calibrated by using the Ag 3d_5/2_ line with a full width at a half-maximum of 1.2 eV. A flood gun operating at 15 μA and 1.5 eV was used to compensate for the charge. The measurements were calibrated against the C 1s peak at a binding energy (BE) of 284.7 eV.

All the samples were prepared for observation by transmission electron microscopy (TEM) by dispersion in toluene followed by sonication for 5 min. A droplet of approximately 2 μL was subsequently deposited on a copper TEM grid covered with a holey carbon film. For morphological characterization, the samples were examined in a Tecnai Spirit TEM operated at 120 kV. The samples were further observed with a JEOL 2010F operated at 200 kV, a Thermo Fisher Scientific Spectra300 operated at 80 kV, and a Thermo Fisher Scientific Talos F200X operated at 200 kV. For TEM imaging of the morphological functionalization of Ag_*x*_Cu_*y*_Se_*z*_ NPs for assessing antibacterial activity (Figure 6A), an HR Fei Talos 200X microscope was used, operated at an accelerating voltage of 100 kV.

Elemental analysis of the sample was conducted through energy-dispersive X-ray spectrometry (EDS), using a molybdenum TEM support grid, to eliminate interference from the conventional Cu grid. Prior to drop casting the NPs diluted in toluene onto the molybdenum grid, an additional cleaning cycle was employed, involving precipitation with ethanol and redispersion in toluene. This process ensures the removal of ligands and prevents contamination under electron beam irradiation. The grid with the deposited sample was treated with argon plasma for 5 s as a cleaning step to minimize the occurrence of contamination.

Both the scanning transmission electron microscopy-high-angle annular dark-field (STEM-HAADF) images and the STEM-EDS elemental mappings were acquired by using a Thermo Fisher Scientific Spectra300 microscope operating at 80 kV. The STEM-HAADF images were acquired with a 0° tilt angle, a spot size of 11, a camera length of 91 mm, a convergence angle of 30 mrad, and a dwell time of 50 μs. The STEM-EDS elemental mappings were obtained using a SuperX G2 model with identical recording settings except for a reduced dwell time (5 μs) to mitigate potential beam damage and contamination during the acquisition time. The subsequent data analysis was carried out using Thermo Fisher Scientific proprietary software, VELOX.

Fast Fourier transforms (FFT) from high-resolution TEM (HRTEM) images and electron diffraction patterns were indexed using Eje-Z in the TEM-UCA software.

The average size and the size distribution of the NPs were obtained by analyzing the TEM micrographs by using ImageJ software.

## Computational Section

For the DFT calculations, the Vienna Ab Initio Simulation Package (VASP)^37,38^ was used, where the projector augmented wave (PAW) method^39,40^ was applied in combination with the generalized gradient approximation (GGA) by Perdew, Burke, and Ernzerhof (PBE).^41^ Settings for the energy cutoff of the electronic wave functions and for the density of the *k*-mesh were tested to ensure energy convergence within 1.0 meV/atom. All structures were calculated using energy cutoffs of 550 eV for the valence electronic wave functions and 770 eV for the augmentation wave functions, respectively. For the metallic AgCuSe phase, a high-density *k*-mesh of 28 × 28 × 20 was used corresponding to a linear *k*-spacing of less than 0.0088 Å^–1^ in any reciprocal lattice direction. For the other compounds, which are semiconductors or insulators, the *k*-meshes were set to have a linear *k*-spacing of less than 0.028 Å^–1^ in any reciprocal lattice direction. Table S1 provides an overview of the calculated phases with the number of atoms per unit cell and the *k*-meshes used in the calculations. During the GGA-PBE calculations, both the cell dimensions and the atomic coordinates were fully relaxed to obtain the lowest energy configurations. Energy convergence criteria of 10^–6^ and 10^–5^ eV were used for the electronic and ionic loop, respectively. DFT calculations are valid for a temperature of 0 K and a pressure of 0 Pa, and spin–orbit coupling effects were not taken into account. These computational settings are the same as the ones used in our previous work.^42^

## Results and Discussion

**Figure 1:**
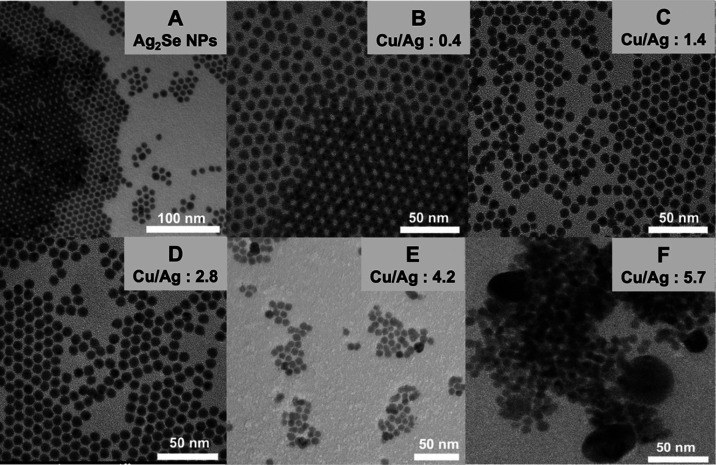
TEM micrographs of (A) Ag_2_Se NPs and samples obtained from the reaction of CuIPPh_3_ with Ag_2_Se NPs with different Cu/Ag precursor molar ratios: (B) 0.4, (C) 1.4, (D) 2.8, (E) 4.2, and (F) 5.7.

**Figure 2:**
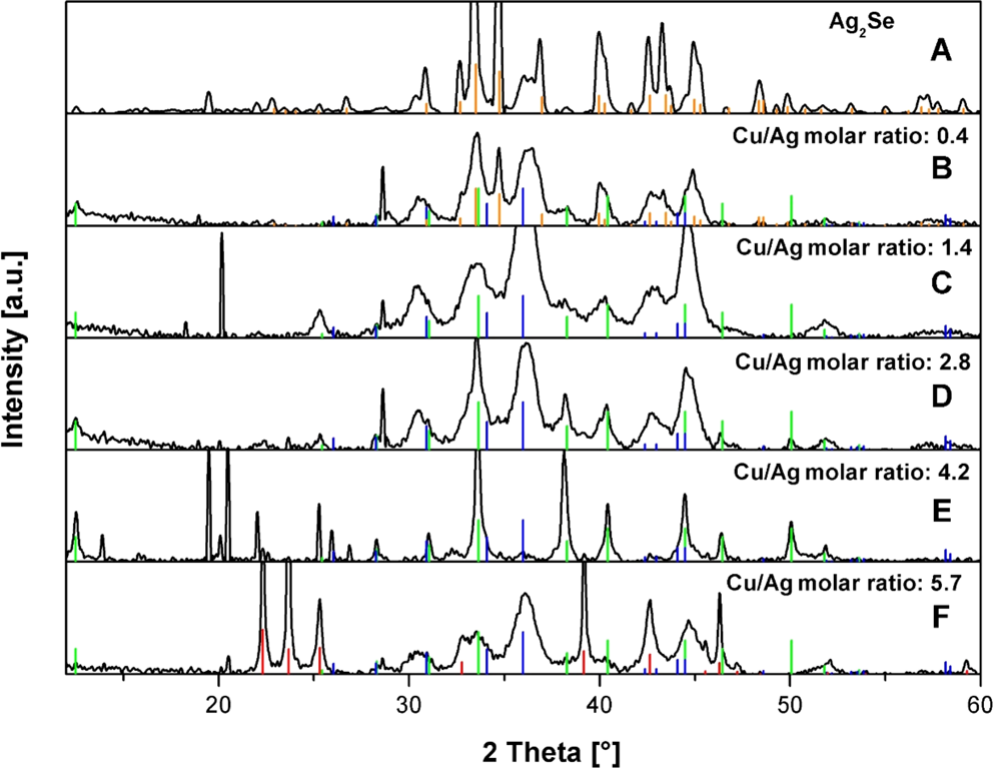
XRD patterns of samples obtained from the reaction of CuIPPh_3_ with Ag_2_Se NPs at (A) 0, (B) 0.4, (C) 1.4, (D) 2.8, (E) 4.2, and (F) 5.7 Cu/Ag precursor molar ratio, AgCuSe (JCPDS 00-089-3935, blue), Ag_3_AuSe_2_ (JCPDS 00- 025-0367, green), Ag_2_Se (JCPDS 00-024-1041, orange), and AgI (JCPDS 01-083-0582, red) reference patterns.

The synthetic process is initiated by preparing monodisperse Ag_2_Se NPs as precursors using the hot-injection method. In Figure 1A, the TEM micrograph illustrates the as-synthesized Ag_2_Se NPs, exhibiting a hexagonal morphology and boasting an average diameter of 7.7 nm with a narrow size distribution (10%). The precursor NPs display a high crystallinity and the expected orthorhombic structure, as shown in Figure 2A.

The synthesis of Ag_*x*_Cu_*y*_Se_*z*_ ternary NPs is realized by mixing Ag_2_Se NPs with a CuIPPh_3_ suspension at room temperature. Figure 1B–F exhibits a series of TEM micrographs illustrating the final NPs obtained at varying Cu/Ag molar ratios. The particle size histograms from Figure 1A–F are presented in Figure S1. Notably, NPs in samples with a Cu/Ag molar ratio below 4.2 exhibit a consistent size and homogeneity akin to the initial Ag_2_Se NPs [(Figure 1A) 7.7 ± 0.7 nm, (Figure 1B) 7.9 ± 0.9 nm, (Figure 1C) 7.6 ± 0.7 nm (Figure 1D) 7.8 ± 0.8 nm]. Conversely, in the sample with the Cu/Ag molar ratio equal to 4.2 (Figure 1E) a slight degree of aggregation is observed with an average size of 7.8 ± 1.1 nm and reduced size homogeneity, which becomes more noticeable in the sample with the highest Cu/Ag molar ratio (Figure 1F), where aggregation of numerous NPs is evident and the presence of some 50 nm-sized NPs is observed. These findings suggest that the further addition of CuIPPh_3_ induces aggregation. Furthermore, NPs obtained with the lowest Cu/Ag molar ratio (0.4, Figure 1B) exhibit an approximately hexagonal shape, whereas those from samples with Cu/Ag molar ratios of 1.4 (Figure 1C) and 2.8 (Figure 1D) display a more cubic morphology with an increased amount of CuIPPh_3_.

Upon analyzing the XRD pattern of the above-mentioned sample with a molar ratio of 4.2 (Figure 2E), the presence of organic crystalline components is suggested by three peaks at around 20°. Low-intensity peaks at 30.9° and 35.9° are tentatively assigned to the (110) and (012) lattice planes of the only reported phase within the Ag–Cu–Se ternary system, namely, *eucairite* with the formula AgCuSe. Additional examination of the peaks reveals three in the range of 22–26° identified as AgI, confirming their status as minor byproducts. Despite most of the peaks remaining unassigned, they do not correspond to either *eucairite* AgCuSe or the initial binary phase of Ag_2_Se. Further analysis of the diffractogram highlights a distinct peak at 12.5°, indicating a relatively large interplanar distance associated with the structure of the ternary *fischesserite* phase, Ag_3_AuSe_2_. This is noteworthy given the absence of Au in our reaction system. Nevertheless, the remaining peaks in the experimental XRD pattern closely align with the reflections attributed to the *fischesserite* reference pattern, particularly those at 12.5, 28.3, 38.3, and 40.4°.

The experimental data strongly suggest the formation of a major phase with a new structure, isostructural to *fischesserite*, specifically Ag_3_AuSe_2_. While the chemical composition of this new structure is not yet confirmed, it is termed Ag_3_CuSe_2_ henceforth, differentiating it from the other reported ternary structure (*eucairite* AgCuSe). In general terms, the coexistence of both ternary phases is denoted as Ag_*x*_Cu_*y*_Se_*z*_

In the XRD pattern of the sample with the lowest Cu/Ag molar ratio (0.4, Figure 2B), the displayed peaks correspond to three crystallographic phases: two ternary phases (AgCuSe and Ag_3_CuSe_2_), as mentioned in the previous paragraph, and the initial orthorhombic phase of Ag_2_Se. The XRD pattern of the sample with the highest Cu/Ag molar ratio (5.7, Figure 2F) exhibits five sharp peaks at 22.3, 23.6, 25.3, 39.1, and 46.2°, attributable to hexagonal AgI, with the remaining peaks associated with AgCuSe and Ag_3_CuSe_2_, although the peaks are relatively broad.

For the other two samples with intermediate Cu/Ag molar ratios (1.4, Figure 2C and 2.8, Figure 2D), their XRD patterns are remarkably similar, revealing only the presence of both ternary phases (AgCuSe and Ag_3_CuSe_2_). Notably, there are no additional crystallographic phases, such as the initial *orthorhombic* phase of Ag_2_Se or hexagonal AgI.

In summary, the identification of the initial Ag_2_Se phase in the sample with the lowest Cu/Ag molar ratio (0.4) suggests that the CE reaction is incomplete, likely due to an insufficient amount of CuIPPh_3_ compound. As the Cu/Ag molar ratio increases, the formation of AgI becomes more favorable: in the XRD pattern of the sample with the highest Cu/Ag molar ratio (5.7), the AgI peaks are relatively narrow, indicating a large microcrystalline size, while the peaks related to the two ternary phases are broad. The formation of the Ag_3_CuSe_2_ ternary phase consistently coincides with the formation of the AgCuSe *eucairite* phase. However, contrary to the expectations, the Cu/Ag ratio is not so critical to selectively promote the crystallization of one ternary compound or the other, which seems to be more sensitive to other experimental conditions yet to be controlled. The XRD patterns of samples with intermediate Cu/Ag molar ratios (Figure 2C–E) suggest these as the most optimal conditions for the formation of ternary Ag_*x*_Cu_*y*_Se_*z*_ nanophases, where apparently the proportion of the new Ag_3_CuSe_2_ phase increases with increasing Cu/Ag molar ratio. Among all tested ratios, the sample with a 2.8 Cu/Ag molar ratio is predominantly composed of two nearly pure ternary phases, devoid of side products such as AgI, organic components, and unreacted binary Ag_2_Se. The sample with a 4.2 Cu/Ag molar ratio exhibits a lower quantity of the AgCuSe phase and a more prominent presence of the optimized Ag_3_CuSe_2_ phase. Notably, the latter phase is particularly intriguing, as it appears to be previously unreported, to the best of our knowledge.

**Figure 3:**
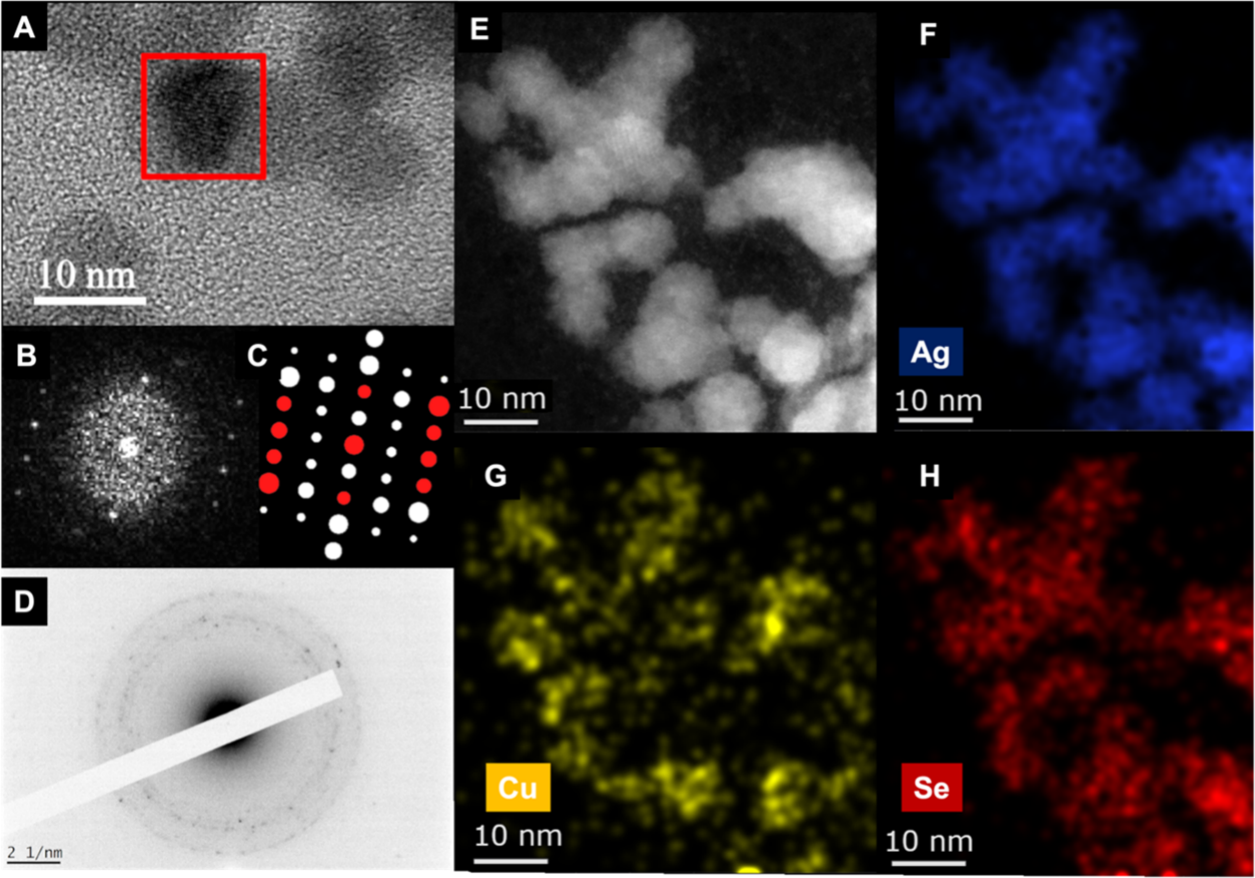
(A) High-resolution TEM micrograph with the FFT of the selected area in (B). (C) [101] *Fischesserite* theoretical electron diffraction pattern, with coincident spots marked in red. (D) Electron diffraction pattern of a group of NPs. (E) STEM-HAADF micrograph of ternary NPs. (F)–(H) the EDS mapping of the same region for Cu, Ag, and Se, respectively.

The sample with 4.2 Cu/Ag molar ratio, prepared at room temperature, undergoes detailed structural and chemical characterization using HRTEM and STEM-EDS. In Figure 3A, an HRTEM micrograph is presented, and the corresponding FFT pattern from the highlighted red square region in Figure 3B is compared with the theoretical diffraction pattern of the *fischesserite* phase from the (101) zone axis, as shown in Figure 3C. All presented reflection spots on the FFT patterns can be indexed in the theoretical diffraction pattern with a (101) zone axis of the *fischesserite* phase, corresponding to the red spots in Figure 3C. None of the spots can be attributed to binary phases nor any of the *eucairite* polymorphs.

Further analysis via electron diffraction was performed with ring patterns from numerous particles, such as the one shown in Figure 3D, thus averaging the information from many crystals. After careful comparison of the measured distances with theoretical values, the closest match was found for a *fischesserite* isostructural phase. The presence of the pseudotetragonal or orthorhombic polymorphs of *eucairite* cannot be ruled out, as the expected reflections would overlap in the electron diffraction pattern. However, only *fischesserite* could account for all the reflections observed.

The elemental mappings reveal the widespread presence of Se and Ag signals throughout the recorded NPs. Concerning the distribution of Cu signals, they are predominantly concentrated in the areas corresponding to NPs, particularly in regions with a brighter contrast (Figure 3E,G). Only a small fraction of the Cu signal is detected outside the NP areas, likely representing spectral noise or originating from unreacted CuIPPh_3_ precursor.

Quantitative elemental composition analyses of the three involved elements are listed in Table 1, presenting their atomic fractions alongside their corresponding error ranges. The experimental values closely align with the theoretical values for Ag_3_CuSe_2_, as presented in the last column of the table. However, considering the coexistence of two ternary phases (AgCuSe and Ag_3_CuSe_2_) in the sample, the chemical analysis can be addressed only qualitatively, not quantitatively. XPS analysis of the same sample (Figure S2) reveals that Cu exists solely in the Cu(I) state (Auger parameter of 1849 eV), Se in the Se(II) state (Se 3d_5/2_ at 54.2 eV), and Ag in the Ag(I) state (Auger parameter of 724.2 eV).

**Table 1:** STEM-EDS Elemental Characterization

element	atomic fraction (atom %) experimental	atomic fraction (atom %) theoretical (Ag_3_CuSe_2_)
Ag–K	41.7 ± 8.0	50.0
Cu–K	18.8 ± 2.2	17.7
Se–K	43.5 ± 8.4	33.3

**Figure 4:**
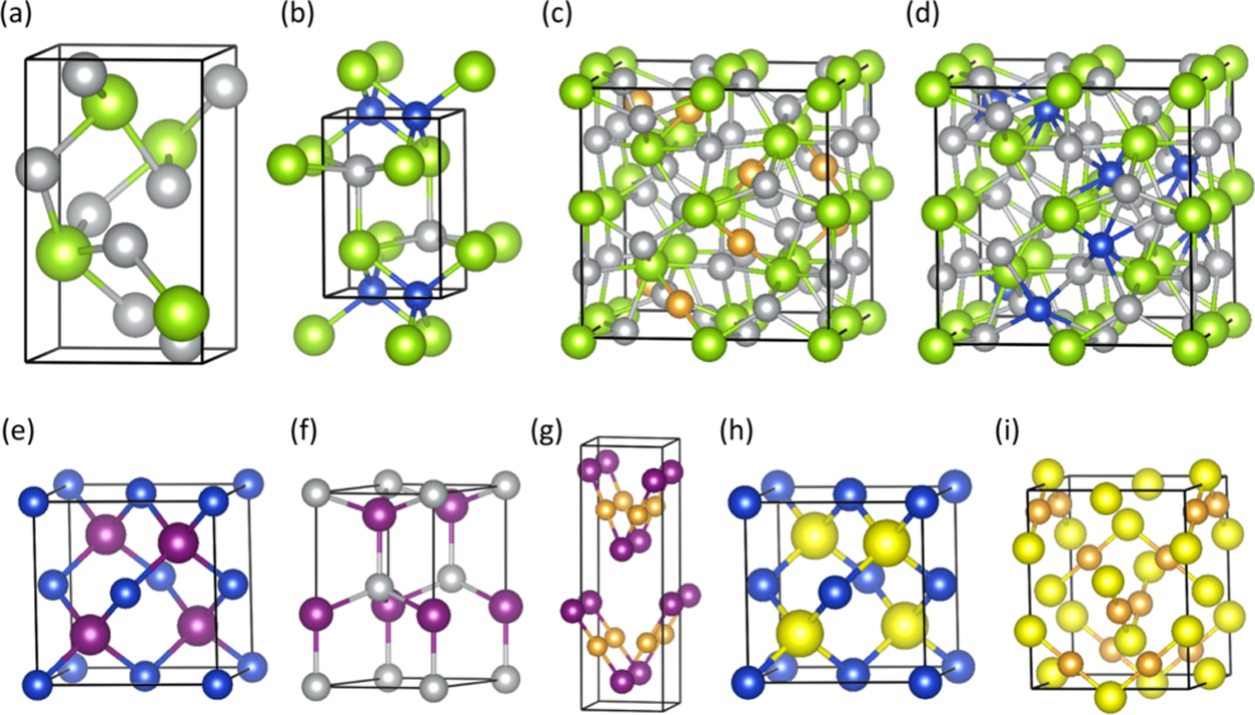
Structural models of M_2_Se, MI, and MCl compounds (M = Ag, Cu, Ag). Silver, gold, blue, green, purple, and yellow spheres depict Ag, Au, Cu, Se, I, and Cl atoms, respectively. The boundaries of the unit cells are indicated with solid black lines: (a) Ag_2_Se; (b) AgCuSe; (c) Ag_3_AuSe_2_; (d) Ag_3_CuSe_2_; (e) CuI; (f) AgI; (g) AuI; (h) CuCl; and (i) AuCl. The phases are given in Table S1. The crystallographic details of the AgCuSe phase are provided in Table S2.

In order to enhance the comprehension of the existence of both ternary phases during the CE reaction between Ag_2_Se and Cu^+^, DFT computations were employed to assess the thermodynamic stability of the phases implicated in the process. The calculations utilized the plane-wave VASP code, with the GGA-PBE functional employed for total energy calculations of fully relaxed structures. Details are provided in the Computational Methods section. Table S1 in the Supporting Information offers an overview of the phases considered in the calculations, while Figure 4 depicts the unit cells of these phases after full relaxation.

AgCuSe occurs as low-temperature *tetragonal* and *orthorhombic* phases, and as a high-temperature *cubic* phase.^24,43^ Here, we have used the tetragonal unit cell for DFT calculations, as this phase is best defined in the literature. The *orthorhombic* phase is very similar to the tetragonal phase; it consists of five unit cells of the tetragonal phase, with small deviations because of a structural feature that repeats itself every five unit cells (it is a superstructure of the *tetragonal* phase). The main complication of the low-temperature AgCuSe phases is that only half the Cu positions are occupied (2 out of 4 equiv sites) and that the Cu atoms are supposedly mobile at room temperature so that the occupation of the positions varies. In XRD experiments, this gives average atomic positions and average occupations, but this variable occupation cannot be applied in DFT calculations, where the atoms are present or not, and where the atoms have zero kinetic energy in total energy calculations. The original publication where the AgCuSe phase was resolved is from 1957^44^ and there is quite some discussion in the literature on the exact structure.^24,43^ In this present work, after having varied the occupation of the Cu sites while relaxing both atomic positions and cell dimensions (shape and volume) of the tetragonal phase, we found a well-converged configuration that agrees well with the experimental lattice parameters. We have used this structure for calculating the total energy of the *tetragonal* AgCuSe phase, and we provide the structural details of this phase in Table S2 of the Supporting Information.

The change in potential energy associated with the various considered CE reactions is provided in Table 2. If the energy change Δ*E* is negative, the DFT calculations predict that there is a thermodynamic driving force for the reaction to take place, while a positive energy change implies that the reaction is unfavorable and will not take place, at least not spontaneously. The energies of reactions 1 and 2 on the right-hand side of Table 2 show that the formation of both AgCuSe and Ag_3_CuSe_2_ from the cation exchange of Ag_2_Se is energetically favorable. When the energy change is expressed per M_2_Se formula unit, it is clear that the formation of AgCuSe is slightly favored over Ag_3_CuSe_2_ (energy gain of 0.194 eV vs 0.173 eV), which agrees with the ubiquitous formation of AgCuSe found in the experiments. Nevertheless, the small difference in energy gain for the formation of the two compounds might likely explain the cocrystallization of the two phases under specific colloidal reaction conditions: the small size of the crystals, high diffusion rates of atoms in solution, and the presence of dynamic surfactants are likely responsible for achieving surface and interface stabilization energies that dictate the coformation and enhance the stability of the less stable *fischesserite*-like phase.

**Table 2:** DFT-Calculated Change in Total Potential Energy Associated with the Listed Reaction Formulas, Expressed Per Reaction Formula and Per M_2_Se Formula unit (M = Ag, Cu, Au)

reaction formula	reaction no.	Δ*E* (eV)	Δ*E* (eV per M_2_Se)
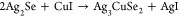 1		–0.346	–0.173
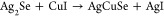 2		–0.195	–0.195
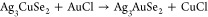 3		–0.002	–0.001
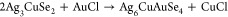 4		–0.358	–0.089

The third reaction, which constitutes full CE from Ag_3_CuSe_2_ to Ag_3_AuSe_2_ after exposure to AuCl, is predicted to have a nearly zero change in potential energy. Thermodynamically, this means that coexistence of the phases is expected. In wet chemistry conditions, this implies that the reaction could be manipulated to take place (by varying, e.g., salt concentrations or solvents). Interestingly, a partial cation exchange from Ag_3_CuSe_2_ to Ag_6_CuAuSe_4_ (reaction (4) in Table 2), where only half the Cu atoms are replaced by Au atoms, is predicted to be energetically favorable, i.e., this reaction is predicted to promote the formation of a relatively stable quaternary (Ag, Cu, Au)_2_Se_1_ phase.

DFT calculations are based on quantum mechanics and are generally considered to be a reliable and self-consistent method for predicting the relative stability of the phases. The main limitation of the DFT method is that the experimental situation often differs from the computational configuration. In particular, here we mention that only bulk phases have been evaluated in the DFT calculations, while at the nanoscale, the (many) surface and interface energies can have an effect on the actual formation of phases. Furthermore, the calculations were performed on chloride and iodide salts in the solid state, while in the experimental situation, the salts are dissolved in solution. The calculated energy differences listed in Table 2 represent the expected thermodynamic driving force for the formation of the phases at the right-hand side of the reaction equations if these would be bulk reactions.

**Figure 5:**
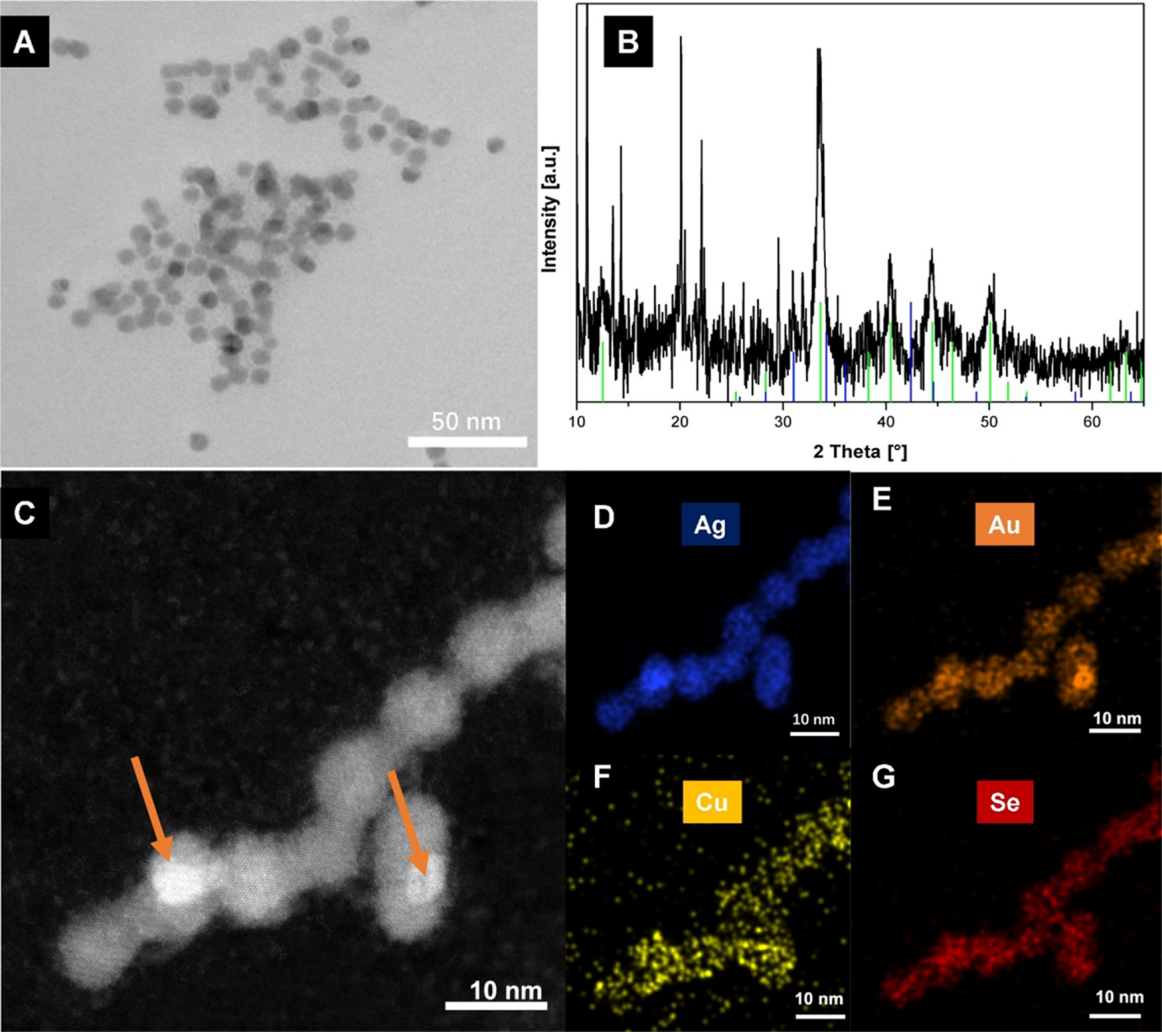
(A) TEM micrograph of NPs obtained after an additional reaction with AuClPPh_3_. (B) XRD patterns of samples obtained after additional reaction with AuClPPh_3_, reference patterns of AgCuSe (JCPDS 00-089-3935, blue), and Ag_3_AuSe_2_ (JCPDS 00-025-0367, green). (C) STEM-HAADF micrograph of the quaternary NPs and from (D)–(G) the EDS mapping of the same region for Au, Cu, Ag, and Se, respectively. (Orange arrows indicate the bright areas).

An additional experiment was designed to authenticate the computational calculation results regarding reaction 3. In the experiment, AuClPPh_3_ solution was added into presynthesized Ag_*x*_Cu_*y*_Se_*z*_ NPs (Cu/Ag = 4.2). The TEM micrograph (Figure 5A) illustrates the obtained NPs, revealing a distinctive pattern: approximately half of the observed NPs exhibit merging, while the remaining half remains isolated, showcasing a roughly spherical shape with an average diameter of approximately 8 nm. In comparison to the TEM micrographs of the initial Ag_*x*_Cu_*y*_Se_*z*_ NPs, very small dots were observed interspersed between the NPs.

Analysis of the XRD pattern of the sample (Figure 5B) indicates the coexistence of two ternary phases, namely, AgCuSe and Ag_3_CuSe_2_. This pattern closely resembles that of the sample containing the ternary initial system, Ag_*x*_Cu_*y*_Se_*z*_. Notably, and contrary to the XRD pattern before the addition of AuClPPh_3_ (Figure 2E), the relative intensities of peaks assigned to the *fischesserite* phase are significantly higher than those assigned to *eucairite* in the sample after adding AuClPPh_3_. This discrepancy may be attributed to the higher thermodynamic stability of the *eucairite* phase (AgCuSe) compared to the Ag_3_CuSe_2_ phase. The *eucairite* phase is the sole reported ternary phase in bulk and the only one found in the crystallographic database, whereas the newly obtained Ag_3_CuSe_2_ phase is likely a metastable phase isolated only at the nanoscale.

Consequently, the cation exchange reaction between Cu(I) in the Ag_3_CuSe_2_ phase and Au(I) from the coordination complex, triggered by the addition of AuClPPh_3_ into the Ag_*x*_Cu_*y*_Se_*z*_ dispersion, likely forms the genuine *fischesserite* phase (Ag_3_AuSe_2_). The structure and composition of the AgCuSe *eucairite* domains remain unaltered. Due to the higher *Z* number of Au in the Ag_3_AuSe_2_ phase compared to that of Cu in the Ag_3_CuSe_2_ phase, the intensity of peaks related to the *fischesserite* phase increases after the CE reaction with AuClPPh_3_.

Chemical characterization of the sample was performed through STEM-EDS, and the elemental mappings in Figure 5D–G reveal the presence of four elements: Au, Cu, Ag, and Se. The Se, Ag, and Au signals appear to be concentrated at the core of the particles, while the Cu energy-filtered image suggests that this element is primarily located at the surface. Additionally, a weak Cu signal is detected in the background. The HAADF image (Figure 5C) shows that Au signals are concentrated in the regions with extremely bright contrast indicated by orange arrows in Figure 5C, suggesting the potential reduction of Au(I) to metallic Au. The quantitative results, presented as atomic fractions in Table 3, indicate a significant drop in the atomic fraction of Cu from 17 atom % in the initial ternary Ag_*x*_Cu_*y*_Se_*z*_ NPs to only 4.7 atom % in the final quaternary structure. As the Cu atomic fraction decreases, the Au atomic fraction rises, becoming the third-highest at 17.6 atom % of the total four elements. Notably, the relative amounts of Ag and Se remain approximately constant after the reaction with AuClPPh_3_.

**Table 3:** STEM-EDS Elemental Characterization Result

element	atomic fraction (%) experimental
Ag–K	38.7 ± 6.8
Cu–K	4.7 ± 0.6
Se–K	39.0 ± 6.9
Au–L	17.6 ± 2.8

These experimental findings align with the earlier assumption of a cation exchange reaction occurring solely between Au(I) from AuClPPh_3_ and Cu(I) from Ag_3_CuSe_2_ NCs in the Ag_*x*_Cu_*y*_Se_*z*_ sample. This induces diffusion of copper atoms/species from the core to the surface of the NPs, ultimately leading to their release. Conversely, Ag(I) cations appeared to remain within the nanostructure.

## Antimicrobial Activity

Metallic Ag NPs are widely recognized for their prominent antibacterial properties.^45^ The antibacterial activity of Ag NPs primarily stems from Ag oxidative dissolution, a process heavily influenced by surface chemistry.^46^ However, bacterial resistance mechanisms against silver are often plasmid-mediated, facilitating the spread of resistance.^47^ Bimetallic NPs including combined metals have been proposed as an alternative,^48^ since the possibility of bacteria developing resistance toward several metals simultaneously is much lower, particularly when different cellular mechanisms are involved.^49,50^ As already stated in the Introduction section, AgCuSe has become the focus of interest in the field of energy conversion during the past decade. However, no studies have been conducted yet to validate their potential in terms of bioactivity. The method reported in this work dictates a protocol for the synthesis of highly homogeneous bimetallic Ag_*x*_Cu_*y*_Se_*z*_ NPs of appropriate sizes for cell adhesion or internalization. In this regard, we explored the antibacterial capacity of Ag_*x*_Cu_*y*_Se_*z*_ NPs.

To analyze the antimicrobial capacity of Ag_*x*_Cu_*y*_Se_*z*_ NPs, those obtained with a precursor molar ratio of 2.8 were selected for testing as the sample comprises only two ternary phases without impurities. However, it was necessary to stabilize them in an aqueous medium. Regrettably, the presence of hydrophobic surfactants such as oleylamine on the NP surfaces did not result in water-stable colloidal NPs. Therefore, to ensure stability in water, a ligand derived from poly(ethylene glycol) (PEG) was synthesized. This ligand was based on a PEG spacer of 1500 Da of molecular weight terminated with a dihydrolipoic acid (DHLA)-derived moiety to bind strongly to the NP surface, and with an amino group. The ligand, DHLA-PEG1500-NH_2_, was prepared in a four-step reaction with good yields (see Supporting Information for more details).^51,52^ Then, the ligand exchange onto the Ag_*x*_Cu_*y*_Se_*z*_ NP surfaces was carried out using a methodology previously reported.^34^

**Figure 6:**
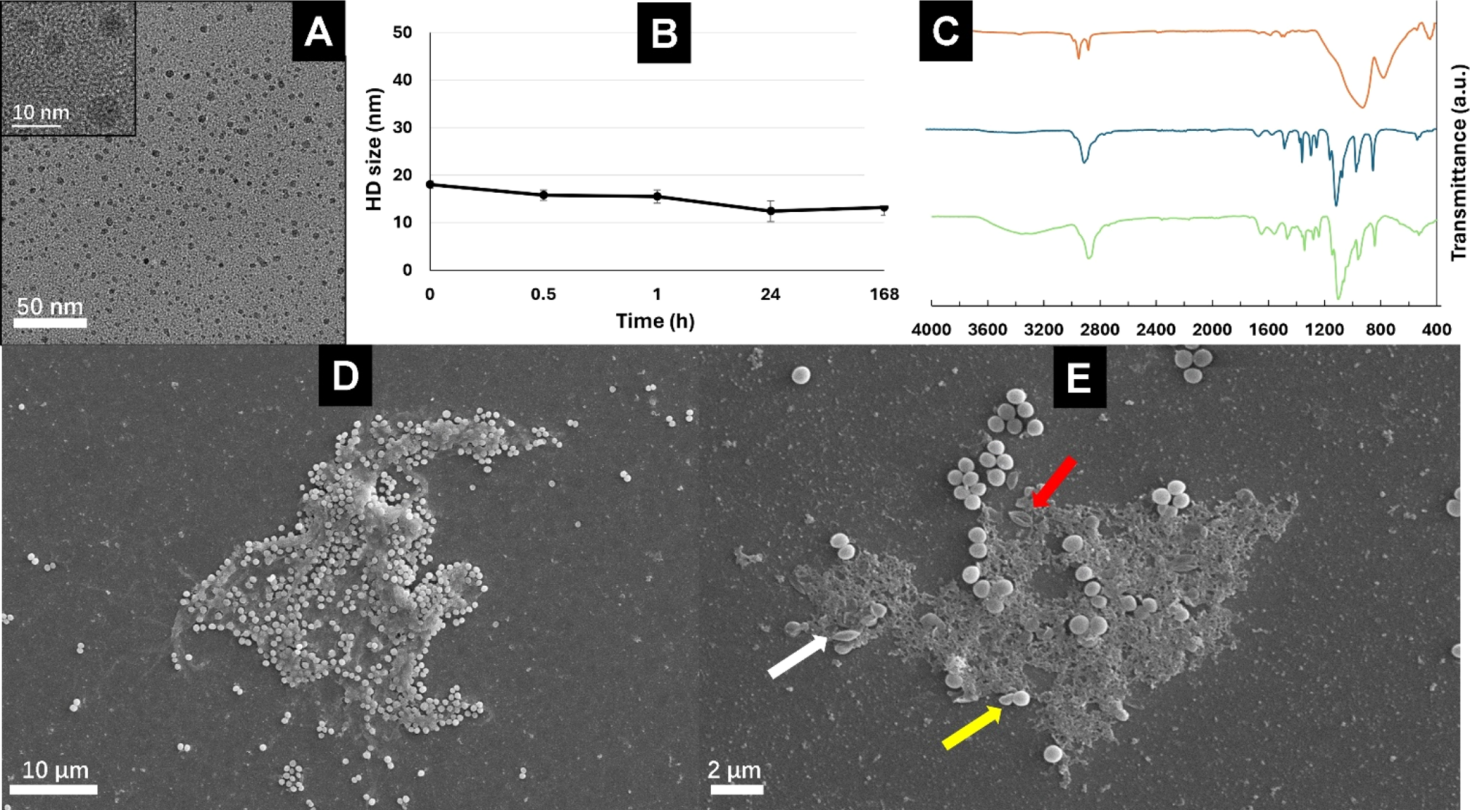
(A) Representative TEM micrograph of PEGylated Ag_*x*_Cu_*y*_Se_*z*_ NPs in water. (B) Hydrodynamic diameter of PEGylated Ag_*x*_Cu_*y*_Se_*z*_ NPs in PBS vs time. (C) FTIR spectra of oleylamine capped Ag_*x*_Cu_*y*_Se_*z*_ NPs (orange line), Ag_*x*_Cu_*y*_Se_*z*_ NPs@DHLA-PEG1500-NH2 (blue line), and ligand DHLA-PEG1500-NH2 (green line). (D) SEM micrograph showing a colony of *S. aureus* formed in the absence of NPs. Note the secretion of extracellular material for attachment and initiation of biofilm formation. (E) SEM micrograph showing the effect of Ag_*x*_Cu_*y*_Se_*z*_ NPs on the morphology and biofilm formation by *S. aureus*. Arrows depict deformed and enlarged cells (white arrow), collapsed cells (red arrow), and cells that were unable to complete proper division (yellow arrow).

The success of the functionalization of Ag_*x*_Cu_*y*_Se_*z*_ NPs was characterized through various techniques, such as TEM, dynamic light scattering (DLS) measurements, and Fourier transform infrared (FTIR) spectroscopy. TEM micrographs (Figure 6A) exhibit an excellent colloidal distribution of the water-soluble PEGylated NPs with no observable aggregates. Furthermore, the colloidal stability of the functionalized NPs was analyzed in phosphate-buffered saline (PBS) over time. As shown in Figure 6B, the functionalized NPs exhibited a hydrodynamic (HD) diameter of 18 nm ±0.8, which remained practically unchanged after 1 week. These results clearly demonstrate the high stability of our PEGylated Ag_*x*_Cu_*y*_Se_*z*_ NPs. FTIR characterization confirmed the presence of DHLA-PEG1500-NH_2_ in the functionalized NPs. As observed in Figure 6C, the FTIR spectrum of the functionalized NPs exhibited peaks practically identical to those of the free ligand. The main peaks in the spectrum can be assigned to C–H stretching (2881 cm^–1^), C=O stretching (1651 cm^–1^), N–H bending (1556 cm^–1^), C–H bending (1466 and 1342 cm^–1^), C–O stretching (1279 and 1240 cm^–1^), and C–O–C stretching (1101 cm^–1^).

To evaluate the antimicrobial capacity, the functionalized Ag_*x*_Cu_*y*_Se_*z*_ NPs were tested against different strains of Gram-positive and Gram-negative bacteria. The results demonstrated excellent antimicrobial activity against Gram-positive *S. aureus*, with a minimal inhibitory concentration (MIC) value of 10 mg of Ag L^–1^ (Table 4). In order to exclude the possibility that the antimicrobial activity was due to the ligand, simultaneous MIC tests were done for PEG1500, dihydrolipoic acid, and the ligand DHLA-PEG1500-NH_2_. In these experiments, the MIC for *S. aureus* for the three substances was >512 mg L^–1^ (results not shown), indicating that there was no inhibition by these compounds. The value obtained is lower than MICs reported for other silver NPs, such as carboxymethyl cellulose stabilized Ag NPs (MIC 60 mg L^–1^) and dendrimer-encapsulated Ag NPs, G5-Ag NPs (128 mg L-1), or similar to lignin-AgNPs (10 mg L^–1^).^53,54^ For the sake of clarity, a comparison of our results with other silver-based NPs and with the MIC for some antibiotics is shown in Table 5. The MIC obtained for our NPs is similar to or even lower than reported for other silver NPs, indicating the usefulness of the NPs synthesized in this work. Moreover, it is higher or comparable to the MIC for several antibiotics.

**Table 4:** Minimal Inhibitory Concentration (MIC) and Minimal Bactericidal Concentration (MBC) of Ag_*x*_Cu_*y*_Se_*z*_ NPs against Gram-positive (*S. aureus*) and Gram-negative (*E. coli* and *P. aeruginosa*) Bacteria

bacteria	MIC (mg of Ag L^–1^)	MBC (mg of Ag L^–1^)
*S. aureus* ATCC 25923	10	40
*Escherichia coli* ATCC 25922	>80	nd
*P. aeruginosa* ATCC 27853	80	nd

**Table 5:** Comparison of the MIC for *S. aureus* for Ag_*x*_Cu_*y*_Se_*z*_NPs with Previously Reported Silver NPs and Antibiotics Usually Employed for this Microorganism

antimicrobial agent	MIC (mg L^–1^)	source
carboxymethyl cellulose stabilized Ag NPs	60	Ambi et al., 2018^56^
dendrimer-encapsulated Ag NPs G5-Ag NPs	128	Dai et al., 2018^54^
lignin-AgNPs	10	Slavin et al., 2021^53^
moxifloxacin^a^	0.25	https://www.eucast.org/clinical_breakpoints
vancomycin^a^	4	https://www.eucast.org/clinical_breakpoints
gentamicin^a^	2	https://www.eucast.org/clinical_breakpoints
tetracycline^a^	1	https://www.eucast.org/clinical_breakpoints
amikacin^a^	16	https://www.eucast.org/clinical_breakpoints
teicoplanin^a^	2	https://www.eucast.org/clinical_breakpoints
Ag_*x*_Cu_*y*_Se_*z*_ NPs	10	this work

a Break points for *S. aureus* published by EUCAST (https://www.eucast.org/clinical_breakpoints).

With regard to MBC, the value obtained for *S. aureus* was 40 mg L^–1^ (in terms of Ag), 4-fold higher than MIC. This means that at 10 mg L^–1^ (MIC) the NPs are acting as bacteriostatic agents, since they inhibit the division of the bacteria, but some of them can still be viable. However, when the concentration increases up to 40 mg L^–1^ the substance acts as a bactericide agent.

It is known that released Ag ions are responsible for the antibacterial activity of most described NPs. In our case, it should be the same situation since we have demonstrated that neither PEG1500, dihydrolipoic acid, nor the ligand DHLA-PEG1500-NH_2_ has antimicrobial activity. The antimicrobial activity of Ag^+^ is due to several mechanisms acting simultaneously, including the production of oxygen reactive species (ROS) such as H_2_O_2_, superoxide, ^•^HO radicals, etc.; the peroxidation of membrane lipids which causes disruption of the bacterial envelope that can produce leaching of substances from the cytoplasm; binding of positively charged Ag^+^ to negatively charged molecules such as proteins or DNA (inhibiting replication); displacement of some metal ions from protein molecules by Ag^+^ (inhibiting enzymatic activity); etc.^55^

Conversely, the values of MIC for Gram-negative bacteria, i.e., *E. coli* and *P. aeruginosa* were 80 and >80 mg of Ag L^–1^, respectively. These higher MIC values made it evident that our Ag_*x*_Cu_*y*_Se_*z*_ NPs could be less effective as antimicrobial agents for Gram-negative strains. This could be attributed to the presence of resistance mechanisms toward Ag, Cu, and Se in Gram-negative bacteria. In fact, operons such as *sil*, which are involved in expelling Ag^+^ and Cu^+^ ions out of the cell, represent one of the most important metal resistance mechanisms found in Gram-negative bacteria.^49^ On the other hand, resistance toward Se is usually accomplished by the reduction of selenate or selenite to Se and the formation of less toxic Se NPs. Some species are capable of volatilizing Se upon the formation of volatile methylated Se species.^50^

SEM was employed to assess the effect of Ag_*x*_Cu_*y*_Se_*z*_ NPs on the morphology and the biofilms of *S. aureus* as well as to preliminarily determine the accumulation of Ag in bacteria. In the absence of NPs, large colonies of cocci were observed (Figure 6D). Moreover, a significant accumulation of extracellular material was noted, indicating strong bacterial adhesion to the glass surface as the initial step in the formation of a dense biofilm.^57^ The extracellular material is composed of a complex mix of polymers, including proteins, exopolysaccharides, and extracellular DNA (eDNA).^58,59^ Biofilms are considered additional virulence factors for nosocomial bacteria, as they promote long-term infections and increase resistance to antibiotics compared to planktonic bacteria.^60^

In the presence of Ag_*x*_Cu_*y*_Se_*z*_ NPs at an MIC of 10 Ag mg L^–1^ (Figure 6E), colonies appeared significantly smaller, accompanied by increased accumulation of extracellular material. In fact, it is known that other silver NPs stimulate matrix production and biofilm formation at sublethal concentrations.^61^ This could be attributed to the protective role of the extracellular matrix against antibacterial chemicals.^62^ Nevertheless, it cannot be excluded that part of this material could be due to the collapse of some of the cells. In this regard, many deformed cells were observed, as indicated by white arrows. Some cells exhibited collapse and a hollow in the cell wall (red arrow), whereas others seemed incapable of completing proper division (yellow arrow). These results suggest a pronounced effect of Ag_*x*_Cu_*y*_Se_*z*_ NPs on cell morphology and physiology. The decrease in biofilm formation at the MIC was also noteworthy, as evidenced by smaller colonies with fewer bacteria. In fact, a minimal bacterial population, a phenomenon known as “quorum sensing” is required for initiation of the biofilm.^63^ Other Ag NPs also demonstrated antibiofilm activity against *E. coli* and *S. aureus*(64) In addition, Se NPs have been described as blockers of biofilms. For instance, biogenic Se NPs produced by *Bacillus mycoides* SelTE01 were able to inhibit biofilm formation by either Gram-positive (*S. aureus*) or Gram-negative (*P. aeruginosa*) bacteria.^65^

Preliminary determination of Ag in the bacteria gave a value of 0.27% of the total weight, measured by EDX and shown in Figure S3. In this regard, metal can be either adsorbed to cells, i.e., bound to the cell wall or to extracellular material including exopolysaccharides, extracellular DNA (eDNA) or extracellular proteins, or absorbed, i.e., accumulated inside the cell.^66^

As a consequence of the environmental release of Ag formulations used in the treatment of infections, the resistance toward this metal in bacteria is increasing.^67^ The most important contribution and difference from other silver NPs is the polymetallic composition. This would prevent or at least dilatate the time for acquisition of resistance by bacteria, since different mechanisms are involved in the resistance toward different metals, so the acquisition of resistance determinants against all the metals is more difficult. Besides Ag, Cu, and Se can also have additional antimicrobial activity against bacteria and fungi.^68,69^ In addition, all the components of the ligand are nontoxic and biocompatible. On the other hand, the release of metal ions from the NPs can provide a slow liberation of these metal ions and maintain a more constant dose of antimicrobial treatment.^68^

## Conclusions

In conclusion, this work presents a comprehensive study, including the synthesis and characterization of AgCuSe ternary NPs via a CE reaction. Our findings reveal distinct morphological changes in the NPs with varying Cu/Ag molar ratios, alongside the formation of a novel ternary phase, being analogous to *fischesserite*, denoted as Ag_3_CuSe_2_. Computational predictions supported experimental observations, enhancing our understanding of phase stability. Furthermore, the functionalization of Ag_*x*_Cu_*y*_Se_*z*_ NPs enabled their dispersion in aqueous media, facilitating the assessment of their promising antimicrobial activity against Gram-positive bacteria, particularly *S. aureus*, and thus becoming more earth-abundant alternatives compared to standard Ag NPs. However, efficacy against Gram-negative strains was comparatively reduced, likely due to inherent resistance mechanisms.

## Supporting Information Available

Ligand synthesis for the antibacterial test, particle size histograms, XPS spectra for Ag*_x_*Cu*_y_*Se*_z_* NPs, EDX spectrum of Ag accumulated in bacteria and parameters for DFT calculation and corresponding results (PDF)
