# Supplementary material for: Unraveling the Formation of Ternary AgCuSe Crystalline Nanophases and Their Potential as Antibacterial Agents
Source: Chem Mater. 2024 Oct 9;36(20):10154–66. doi: 10.1021/acs.chemmater.4c01604 (PMC11500304; doi:10.1021/acs.chemmater.4c01604)
Supplement: Supplementary file 1 — cm4c01604_si_001.pdf [file cm4c01604_si_001.pdf]

Supporting Information

Unraveling the formation of ternary AgCuSe crystalline nanophases and their potential as antibacterial agents

Mengxi Lin, Beatriz Vargas, Lluís Yedra, Heleen van Gog, Marijn A. van Huis, Rafael G. Mendes, Jordi Llorca, Manel Estruch-Blasco, Manuel Pernia Leal, Eloísa Pajuelo, Sònia Estradé, Francesca Peiró, Laura Rodríguez, Albert Figuerola*.

Supporting experimental section

Ligand synthesis

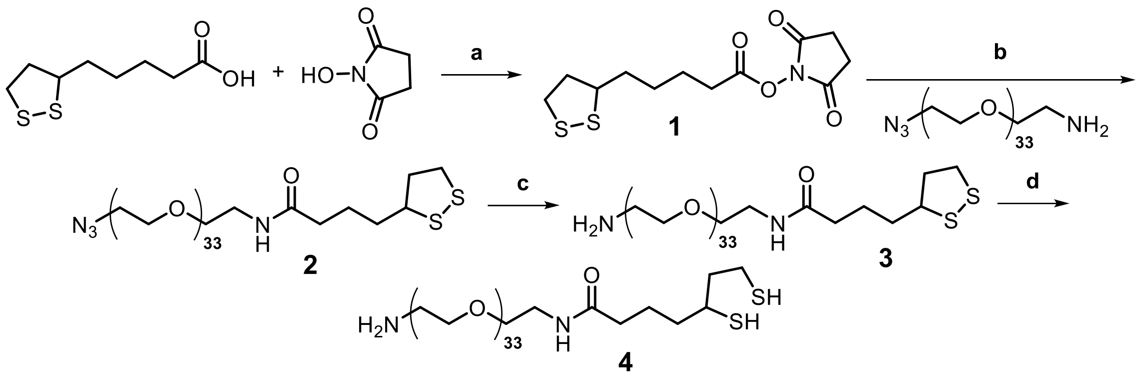

Scheme S1. a) N,N'-dicyclohexylcarbodiimide (DCC), CH_2Cl_2, 0 °C to room temperature (r.t.), 1 h, 73%. b) CH_2Cl_2, 2 h, 76%. c) PPh_3, Tetrahydrofuran, overnight, 85%. d) $NaBH_4$, EtOH, H_2O, 0 °C to r.t., 4 h, 70%.

Compound **1**

The synthesis was done following a procedure already described in the literature. (Shi, L, 2013) Briefly, to a solution of lipoic acid (500 mg, 2.42 mmol) and N-hydroxysuccinimide (306 mg, 2.66 mmol) in 4mL of dry CH_2Cl_2 in a 50 mL round-bottom flask at 0 °C under an Ar atmosphere was added dropwise a solution of N,N'-dicyclohexylcarbodiimide (624 mg, 3.02 mmol) in 3 mL of dry CH_2Cl_2. The mixture was stirred at room temperature (r.t.) for 1 h. After that, the resulting yellow solution was cooled at -20 °C overnight and filtered to remove the precipitated urea as a white solid. Then, the product was precipitated from the supernatant with Et_2O, filtered and vacuum dried. The activated acid was obtained as a pale yellow solid (603 mg, 1.99 mmol, 73%) and was used without further purification. R_f =0.9 (CH_2Cl_2/CH_3OH, 15:1). ^1H NMR (300 MHz, $CDCl_3$): δ (ppm) 3.66–3.51 (m, 1H), 3.26–3.04 (m, 2H), 2.84 (br s, 4H), 2.63 (t, *J* = 7.3 Hz, 2H), 2.55–2.39 (m, 1H), 1.99–1.87 (m, 1H), 1.86–1.73 (m, 2H), 1.76–1.67 (m, 2H), 1.67–1.47 (m, 2H).

Compound **2**

To a solution of compound **1** (429 mg, 1.42 mmol) in 5 mL of dry CH_2Cl_2 in a 25 mL round-bottom flask under an argon atmosphere was added a solution of the amino-PEG1500-N_3 (1.97 g, 1.29 mmol) in 10 mL of dry CH_2Cl_2. The mixture was stirred at r.t. for 2 h. After that, 5 mL of HCl 1M was added to the mixture and the phases were separated. The aqueous phase was extracted with CH_2Cl_2 (2 x 10 mL) and the combined organic phases were dried over anhydrous Na_2SO_4. The product was precipitated with Et_2O at -20 °C as a low melting point white solid (1.98 g, 1.16 mmol, 76%). R_f =0.45 (CH_2Cl_2/CH_3OH, 15:1). ^1H NMR (300 MHz, $CDCl_3$): δ (ppm) 6.23 (br s, 1H), 3.89–3.31 (m, PEG, 1 H), 3.22–3.01 (m, 2H), 2.51–2.35 (m, 1H), 2.16 (t, *J* = 7.4 Hz, 2H), 1.97–1.80 (m, 1H), 1.74–1.56 (m, 4H), 1.53–1.37 (m, 2H).

Compound **3**

Compound **2** (1.98 g, 1.16 mmol) and triphenylphosphine (509 mg, 1.94 mmol) were dissolved in 50 mL of dry tetrahydrofuran (THF) in a 100 mL round-bottom flask under an argon atmosphere. The mixture was stirred at r.t. for 2h. Next, 1 mL of H_2O was added to the mixture, and it was stirred overnight. THF was evaporated and, EtOAc (30 mL) and HCl (20 mL, 1M) were added. The phases were separated, and the aqueous phase was then washed with EtOAc until the phosphines were removed. Na_2CO_3 was slowly added to the aqueous phase until pH ≈ 9 and the product was extracted several times with $CHCl_3$. The combined organic phases were dried over anhydrous Na_2SO_4 and evaporated under reduced pressure. The product was obtained as a white wax (1.66 g, 0.99 mmol, 85%). R_f =0.3 (CH_2Cl_2/CH_3OH, 15:1). ^1H NMR (300 MHz, $CDCl_3$): δ (ppm) 6.16 (br s, 1H), 3.93–3.33 (m, PEG, 1H), 3.24–3.03 (m, 2H), 2.88 (t, *J* = 5.2 Hz, 2H), 2.53–2.36 (m, 1H), 2.22 (br s, 2H) 2.18 (t, *J* = 7.4 Hz, 1H), 1.98–1.81 (m, 1H), 1.74–1.60 (m, 4H), 1.53–1.40 (m, 2H).

Compound **4**

To a solution of compound **3** (1.64 g, 0.97 mmol) in 20mL of EtOH in a 50 mL round-bottom flask at 0 °C under an argon atmosphere was added dropwise a solution of $NaBH_4$ (150.5 mg, 3.90 mmol) in 3 mL of H_2O. The mixture was stirred at r.t. for 4 h and the solvent was evaporated under reduced pressure. The residue was dissolved in 20 mL of CH_2Cl_2 and was washed with 10 mL of brine. The organic phase was dried over anhydrous Na_2SO_4, and evaporated under reduced pressure. The product was obtained as a white wax (1.14 g, 0.68 mmol, 70%). R_f =0.3 (CH_2Cl_2/CH_3OH, 15:1). ^1H NMR (400 MHz, $CDCl_3$): δ (ppm) 6.25 (br s, 1H), 3.85–3.39 (m, PEG), 3.39–3.30 (m, 1H), 3.22–3.05 (m, 2H), 2.89 (t, *J* = 5.2 Hz, 2H), 2.50–2.40 (m, 1H), 2.18 (br s, 2H), 1.96–1.83 (m, 1H), 1.76–1.57 (m, 4H), 1.53–1.39 (m, 2H).

Supporting Tables

Table S1. Overview of DFT-calculated M–Se, M-I, and M-Cl (M=Ag,Cu,Au) systems. The lattice parameters are obtained after full relaxation of both cell dimensions and atomic positions.

System	Compound	Structure	Atoms per unit cell	k-mesh	Lattice parameters
M–Se	Ag_2Se	Naumannite	12	6×8×8	*a*=4.526 Å, *b*=7.080 Å, *c*=7.690 Å
	AgCuSe	tetragonal	6	28×28×20	*a*=4.163 Å, *c*=6.253 Å
	Ag_3CuSe_2	Fisschesserite	48	4×4×4	*a*=10.048 Å
	Ag_3AuSe_2	Fisschesserite	48	4×4×4	*a*=10.109 Å
M–I	CuI	zinc blende	8	6×6×6	*a*=6.083 Å
	AgI	wurtzite	4	8×8×6	*a*=4.692 Å, *c*=7.675 Å
	AuI	tetragonal	8	10×10×4	*a*=4.604 Å, *c*=14.625 Å
M-Cl	CuCl	zinc blende	8	8×8×8	*a*=5.429 Å
	AuCl	tetragonal	16	6×6×6	*a*=7.185 Å, *c*=8.767 Å

Table S2. Structural details of the low-temperature tetragonal AgCuSe phase as obtained from the DFT calculations after full relaxation. To avoid confusion on the partial occupation of Cu sites (see main text), here we provide all atomic coordinates. Please note that after our relaxation, the Cu atoms occupied high-symmetry 2a Wyckoff sites (z_{rel}=0) rather than 4f Wyckoff sites (z_{rel}≠0). Experimental lattice parameters from Ref. [Frueh1956] are included for comparison.

Space group	P4/*mmn* (no. 129)		
Lattice parameters	This work		Literature (exp.)
a (Å)	4.163		4.07
b (Å)	4.163		4.105
c (Å)	6.253		6.31
Nr of atoms in unit cell	6		
Atomic positions			
Atom	Wyckoff site	(x_{rel},y_{rel},z_{rel})	
Cu1	2a	(¼; ¾; 0)	
Cu2	2a	(¾; ¼; 0)	
Ag1	2c	(¼; ¼; 0.6922)	
Ag2	2c	(¾; ¾; 0.3078)	
Se1	2c	(¼; ¼; 0.2506)	
Se2	2c	(¾; ¾; 0.7494)	

Supporting Figures

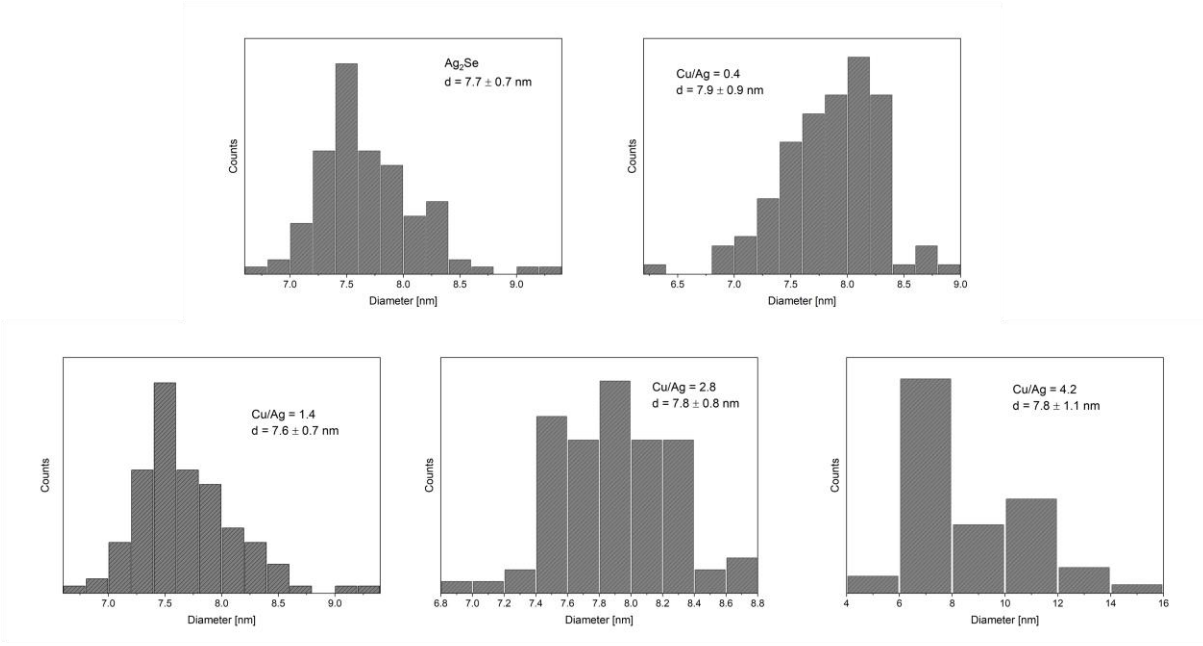

Figure S1. Paticle size histograms of Ag_2Se NPs and samples with different Cu/Ag ratios.

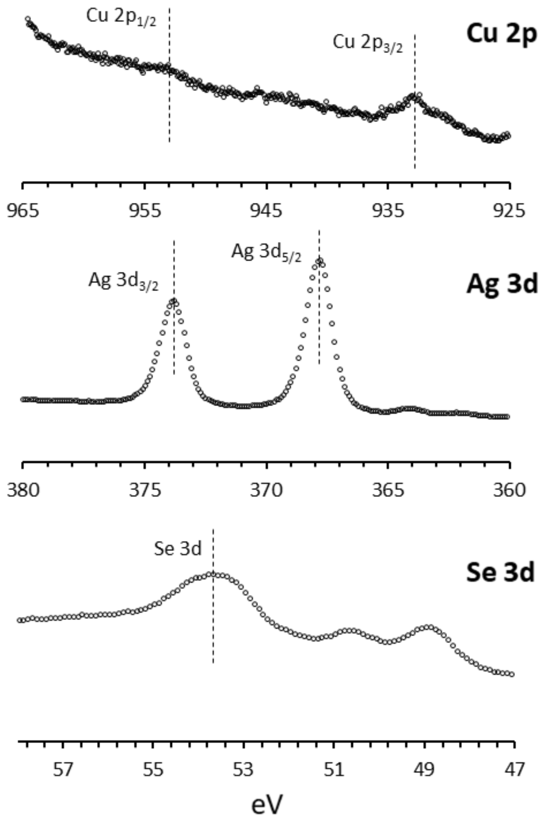

Figure S2. Cu 2p, Ag 3d and Se 3d photoemission spectra (XPS) of the AgCuSe ternary sample.

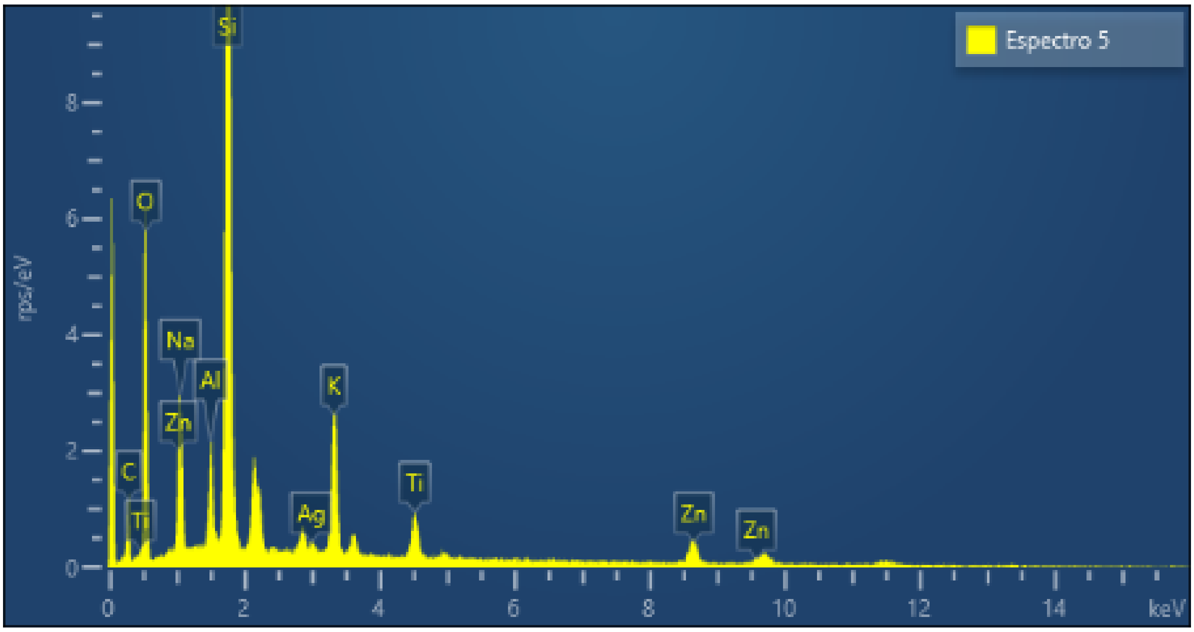

Figure S3. EDX spectrum of Ag accumulated in bacteria.
